# Title and Abstract Screening and Evaluation in Systematic Reviews (TASER): a pilot randomised controlled trial of title and abstract screening by medical students

**DOI:** 10.1186/2046-4053-3-121

**Published:** 2014-10-21

**Authors:** Lauren Ng, Veronica Pitt, Kit Huckvale, Ornella Clavisi, Tari Turner, Russell Gruen, Julian H Elliott

**Affiliations:** 1 Faculty of Medicine, Nursing and Health Sciences, Monash University, Melbourne, VIC 3168, Australia; 2 National Trauma Research Institute, 85-89 Commercial Rd, Melbourne, VIC 3004, Australia; 3 Global eHealth Unit, Imperial College London, South Kensington Campus, London SW7 2AZ, UK; 4 World Vision Australia, 1 Vision Drive, Burwood East, Melbourne, VIC 3151, Australia; 5 Department of Infectious Diseases, Alfred Hospital and Monash University, 2nd Floor, Burnet Tower, Alfred Hospital, Commercial Road, Melbourne, VIC 3004, Australia; 6 Australasian Cochrane Centre, School of Public Health and Preventive Medicine, 99 Commercial Road, Melbourne, VIC 3004, Australia

**Keywords:** Systematic review, Citation screening, Title and abstract screening, Medical students, Methodology, Technology, Randomised controlled trial, Clinical practice guideline

## Abstract

**Background:**

The production of high quality systematic reviews requires rigorous methods that are time-consuming and resource intensive. Citation screening is a key step in the systematic review process. An opportunity to improve the efficiency of systematic review production involves the use of non-expert groups and new technologies for citation screening. We performed a pilot study of citation screening by medical students using four screening methods and compared students’ performance to experienced review authors.

**Methods:**

The aims of this pilot randomised controlled trial were to provide preliminary data on the accuracy of title and abstract screening by medical students, and on the effect of screening modality on screening accuracy and efficiency. Medical students were randomly allocated to title and abstract screening using one of the four modalities and required to screen 650 citations from a single systematic review update. The four screening modalities were a reference management software program (EndNote), Paper, a web-based systematic review workflow platform (ReGroup) and a mobile screening application (Screen2Go). Screening sensitivity and specificity were analysed in a complete case analysis using a chi-squared test and Kruskal-Wallis rank sum test according to screening modality and compared to a final set of included citations selected by expert review authors.

**Results:**

Sensitivity of medical students’ screening decisions ranged from 46.7% to 66.7%, with students using the web-based platform performing significantly better than the paper-based group. Specificity ranged from 93.2% to 97.4% with the lowest specificity seen with the web-based platform. There was no significant difference in performance between the other three modalities.

**Conclusions:**

Medical students are a feasible population to engage in citation screening. Future studies should investigate the effect of incentive systems, training and support and analytical methods on screening performance.

**Systematic review registration:**

Cochrane Database CD001048

## Background

The continuing exponential growth in published biomedical research presents a daunting challenge for clinicians and others involved in health care [1]. It is increasingly difficult for health care decision-makers to find and appraise research evidence, leading to lost opportunities to translate research investment into health care practice improvement [2] and reducing their utility for clinicians and policymakers. Systematic reviews that collate a body of literature present a high quality resource for health care decision-making but involve a significant investment in time and effort, usually by small groups of skilled individuals.

A key step in the process of systematic review is citation screening, which involves manual review of study report titles and abstracts to identify potentially eligible articles for inclusion in the review [3]. Citation screening is time-consuming yet a crucial aspect of the systematic review process, since failure to identify relevant studies can jeopardise the validity of a review. In order to increase the reliability of article selection, the use of two reviewers has been recommended [4], but this increases the resource requirements of review production.

One approach to improving the timeliness and efficiency of systematic review production in order to maximise its relevancy to clinicians and policymakers is to extend the population of contributors beyond traditional review authors. By engaging a broader community in systematic review production, the total pool of available human resource for review production is increased. This may lead to efficiency gains and is consistent with the efforts to broaden the involvement of stakeholders in the production and use of evidence synthesis. Whilst previous studies have assessed the effect of experience on the accuracy of data extraction [5], there is no empirical evidence regarding the performance of non-expert groups in citation screening or the optimal approaches to support their contributions. To be feasible, methods will be needed to engage and train these screeners, allocate workload, support a decentralised screening process and subsequently aggregate individual screening decisions.

Medical students may be an appropriate group to engage in citation screening for systematic reviews in health care. Medical training confers domain-specific knowledge, and students show interest in getting involved in ‘real world’ academic projects. To explore the feasibility of engaging students in citation screening, we compared the performance of student screeners against expert judgments for a single review. We hypothesised that the modality or technology used for screening may effect the accuracy or efficiency and therefore compared student screening performance using four alternative screening modalities.

## Methods

### Study design

The objectives of the Title and Abstract Screening and Evaluation in Systematic Reviews (TASER) trial were to provide preliminary data on the accuracy of medical student title and abstract screening and on the effect of screening modality on screening accuracy and efficiency. Medical students were randomly allocated to title and abstract screening using one of the four modalities.

We restricted the scope of our study to title and abstract screening using a uniform approach to the whole set of citations as this is the most common approach to citation screening. We did not include screening of full-text articles, the second step in study identification, as the characteristics of this task differs substantially from citation screening.

### Participant eligibility and recruitment

Students enrolled in a Bachelor of Medicine and Surgery at Monash University, Melbourne, Australia in third year and above, or undertaking a research year as part of a Bachelor of Medical Science were eligible for inclusion in the study. All students had received some training in the development of critical appraisal skills within the first 2 years of their study but did not have any previous experience in undertaking systematic reviews. The Monash University Faculty of Medicine distributed an invitation email to a convenience sample of the 1,148 eligible medical students with an explanatory statement to eligible participants using student email lists. All student participants were required to have access to an iOS device (iPhone, iPod Touch or iPad), in addition to a computer with Internet access. Students were offered a double movie ticket and a certificate of participation for their involvement. The study ran from June to August 2012.

### Screening modalities

The four screening modalities included in this study were the following:

EndNote X5 [6], a computer-based reference management software program in which students clicked on each citation to indicate whether the citation was assessed as potentially relevant or not.

Paper, printed with titles and abstracts in a list and students highlighted relevant articles.

ReGroup [7], a web-based systematic review platform, which presents titles and abstracts in a list view. Users click on buttons placed next to each citation to indicate whether they have assessed the citation as potentially relevant or not.

Screen2Go [8], an iOS mobile citation screening application. Citations are presented to the user on the screen of the mobile device and they click on a button to indicate whether the citation is a potentially relevant study or not.

### Randomisation and training

A randomisation schedule was created using Microsoft Excel (Microsoft Corp., Redmond, WA, USA) by an independent investigator. Participants were randomised 1:1:1:1 to one of the four study arms according to the concealed randomisation schedule, as they responded to the invitation email. All participants were emailed with details of how to access their randomised screening modality and a one-page summary (refer to Additional file 1) of the systematic review protocol.

Students in the hard copy print-out group received the citations via post and were provided with highlighters to allocate citations. The EndNote and Screen2Go groups required specific downloading instructions to access the screening programs. EndNote X5 was accessible to students through the Monash University Library website. Screen2Go was undergoing beta testing during the study and required students to download two applications: an application to manage the test user installation process and the Screen2Go application itself. Students allocated to ReGroup each received an individualised email with a web link to register an account. No further support was provided other than the one-page summary of the systematic review protocol with inclusion criteria.

### Citation dataset and screening

A set of citations retrieved from a search conducted for the purpose of updating a single Cochrane systematic review, ‘hypothermia for traumatic brain injury’ [9], was used as the citation dataset. Six hundred fifty citations were obtained from the date of last search from 6 April 2009 until 12 May 2012.

Study participants were asked to screen the full citation set and, for each citation, decide whether it should be included or excluded from the review using a one-step process. Participants were instructed to include citations if, based on the title and abstract, it appeared to meet all the inclusion criteria of the systematic review protocol (Table 1). Participants were given an additional option of marking any citation falling broadly within the parameters of the review or containing insufficient information to make a firm decision as ‘unsure’.

**Table 1:** Inclusion criteria in systematic review protocol

**Parameters**	**Description**
Type of studies	All randomised controlled trials of mild hypothermia versus control (open or normothermia) will be included
Types of participants	Patients with any closed head injury requiring hospitalisation
Types of interventions	Therapeutic cooling, either locally or systemically, by means of a fluid-filled cooling blanket, a ‘bear-hugger’ air-cooling device, ice water lavage, any combination of the above, or other methods, to a target temperature of at most 34–35 degrees Celsius for a period of at least 12 h

### Study outcomes

The primary outcomes of the study were the sensitivity and specificity of participant screening decisions compared to the screening decisions by two independent experienced systematic reviewers. Sensitivity indicates the ability of participants to correctly identify definitively relevant citations whilst specificity indicates minimising the inclusion of irrelevant citations that an expert reviewer would exclude at screening.

Sensitivity (‘final sensitivity’) was defined as the number of citations deemed relevant by the experienced reviewers (included in the final set of studies for the review update after both screening and full-text review) that were correctly identified by the student screener (true positives), divided by the number of true positives plus the number of citations included in the final set of studies by the experienced reviewers that were not included by the student (false negative). The screening specificity of participant screening decisions was defined as the number of citations excluded by the student that were also excluded from the final set of studies by the expert reviewers (true negative), divided by the number of true negatives plus the number of citations included by the student that were not deemed relevant by the experienced reviewers after both screening and full-text review (false positive).

Secondary outcomes were full-text burden (a measure of workload; the proportion of all citations kept for full-text review at the completion of screening) and the total time taken to screen the full citation set by medical student participants.

### Statistical methods

We performed a complete case analysis of all participants who completed screening. We used a chi-squared test [10] to compare completion rates in each of the four modalities. Screening decisions were dichotomised for analysis by collapsing ‘include’ and ‘unsure’ assignments into a single category since, in practice, both would be carried forward for further consideration after title and abstract screening.

Screening specificity and burden were calculated for each participant from the confusion matrix of their screening decisions against the consensus screening decisions of the expert reviewers. Final sensitivity was calculated in a similar way against those articles ultimately selected for inclusion into the review update.

Simple descriptive statistics was used to summarise the distribution of observed performance by screening modality. Data were summarised using box-and-whisker plots with whiskers denoting minimum and maximum observed values, boxes delineating quartile ranges and, separately, data points indicating within-group means. Anticipating negative skew, particularly for specificity, we used non-parametric methods for statistical comparisons of screening modalities. For each of the primary outcomes, we used a Kruskal-Wallis rank sum test [11] to compare all four modalities simultaneously. If a between-modality difference was seen for a particular outcome, we compared each against the EndNote modality representing current standard practice using the Mann-Whitney U test [12]. To compensate for multiple comparisons, we used a sequential Bonferroni correction [13] to adjust a pre-specified significance level of 5%. Because the magnitude of this correction differs for each comparison, we report the effective required significance level in addition to the *p* value that was obtained. If the Kruskal-Wallis test showed no significant difference between groups, then pairwise comparisons were not performed.

Participants in the EndNote, ReGroup and Paper screening groups were requested to self-report the time taken to screen the 650 citations. The Screen2Go program recorded time taken directly by tracking the time during which the software was being used for screening by participants. We examined the relationship between final sensitivity and screening time for the Screen2Go objective timings by calculating the Spearman’s rank correlation coefficient [14].

### Ethics approval, consent and registration

Ethics approval for the project was obtained from the Monash University Human Research Ethics Committee (CF 12/1398-2012000738). Participants were provided with an explanatory statement detailing the study and its purpose. Response to the invitation email and completion of citation screening implied participant consent. The study followed an *a priori* protocol and was not registered as there were no patient participants.

## Results

**Figure 1:**
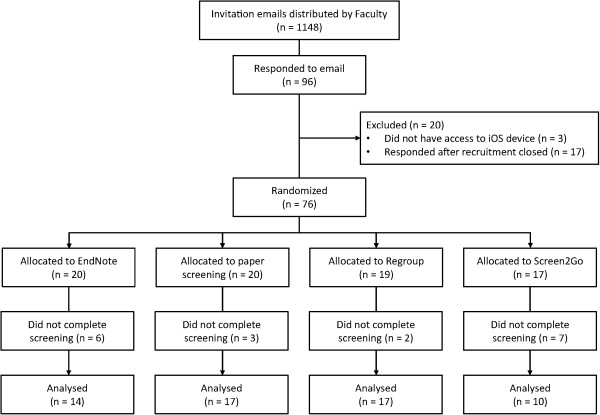
CONSORT flow diagram showing the recruitment processes and losses at each stage.

A total of 76 students were randomised into four screening groups (Figure 1). Baseline demographic data was not obtained. Eighteen participants did not complete screening and were not included in the analysis, leaving 58 participants with evaluable data. The proportion of participants completing citation screening did not differ between study arms (*p* =0.113).

### Primary outcomes: final sensitivity and screening specificity

**Figure 2:**
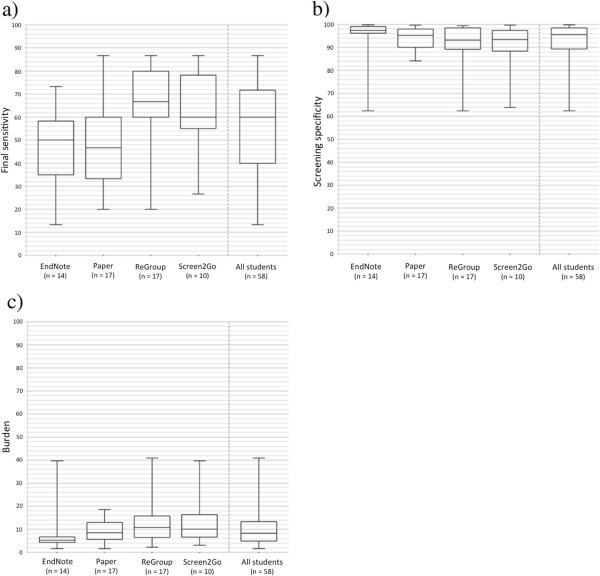
**Student screening performance by modality.** Plots **(a)** and **(b)** show the distribution of final sensitivities **(a)** and screening specificities **(b)** observed in each screening modality as well as the overall pooled distribution, using those study reports ultimately retained in the review update after full-text review as the reference standard. Panel **(c)** shows the burden for each modality calculated using those study reports retained at screening by expert reviewers as the reference standard. Burden is a measure of workload that captures the proportion of all citations that need to be reviewed once screening is completed. Whiskers represent the minimum and maximum values and boxes delimit quartile ranges. The centre line in each box is the median value.

**Figure 3:**
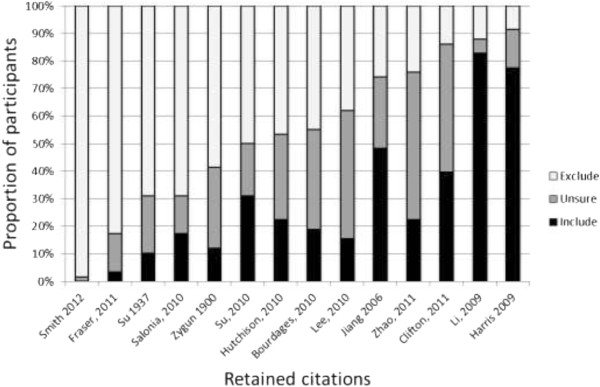
**Retrospective analysis of participant screening decisions for review authors’ final included citations.** The figure summarises the distribution of responses across all students against the 14 citations that were ultimately retained into the review update after review of full text by the expert reviewers.

Within-group median final sensitivity ranged from 46.7% to 66.7% (Figure 2a) and median screening specificity ranged from 93.2% to 97.4% (Figure 2b). Participants in the ReGroup modality demonstrated the highest median sensitivity and the lowest specificity. Kruskal-Wallis tests indicated a significant difference between at least one modality for final sensitivity (*p* =0.015) but not for specificity (*p* =0.147). We therefore conducted pairwise comparison of sensitivity, but not specificity, between the EndNote modality and the other methods. Compared to the EndNote modality, ReGroup sensitivity was significantly higher (*p* =0.005, required significance level after correction =0.017). Differences between Paper and EndNote (*p* =0.689, significance level =0.05) and Screen2Go and EndNote (sensitivity *p* =0.064, significance level =0.025) were non-significant.The highest sensitivities and specificities observed in any single participant were 86.7% and 99.8%, respectively. No participant identified all 14 articles selected for inclusion in the review. Figure 3 illustrates the variability in student screening decisions for each of these articles. The numbers of students correctly selecting each study ranged from 1 (2%, for Smith 2012) to 53 (91%, for Harris 2009).

### Screening burden

Screening burden varied from 5.3% to 10.8% (Figure 2c). No significant difference was seen between groups (*p* =0.053).

### Total screening time

Students in the EndNote, Paper and ReGroup screening groups had median (range) self-reported total screening times of 3 h 30 min (range 01:50–08:00), 3 h 7 min and 30 s (range 02:20–06:41) and 3 h 30 min (range 02:50–07:50), respectively, equivalent to median screening times of 19 (range 10–40), 17 (range 12–37) and 19 s (range 13–41) per citation. Screen2Go captured objective timing information but included safeguards to discard timings when it was unclear if the device was being used for screening or not. As a result, complete timing information was available for only seven of the ten participants in this modality. Median screening time was 3 h 1 min (range 01:15–05:27), equivalent to 17 s (range 7–30) per citation. Spearman’s rho for rank correlation between final sensitivity and total screening times showed a weak positive correlation (rho =0.342) not significantly different from 0 (*p* =0.452).

## Discussion

We performed a pilot randomised controlled trial to compare the performance characteristics of systematic review citation screening by medical students utilising four different screening modalities. Overall, student performance was highly variable and below that of experienced review authors. The use of ReGroup, a web-based systematic review platform, was associated with improved final sensitivity and lower screening sensitivity. Time to screen 650 citations was also highly variable, but did not differ significantly between groups and was not associated with screening sensitivity. There were no other significant differences between groups.

There are several potential reasons for the modest and variable overall performance of medical students’ ability to select relevant articles in this study, when compared to the review authors’ decisions. Firstly, we trialled a minimalist and easily scalable model where students did not receive any training in citation screening as part of the study. All students had received some general training in evidence-based medicine during their medical course, but this did not include specific training in the conduct of systematic review. Future work should explore the effect of different forms of training and support on participant performance. Secondly, participants were given very little guidance in the content area of the review, other than a one-page review protocol with inclusion criteria. Students may have received some previous training in the management of traumatic brain injury, but the gap between their knowledge and that of the review authors is likely to have been substantial. Furthermore, the study was conducted on an update to a review that the review authors had originally conducted, which may have contributed to their expertise on the specific topic of the review. Thirdly, incentives for participation were not linked to the quality of screening decisions. This may have encouraged students to complete the screening as quickly as possible, without regard to the accuracy of their decisions. For example, the title and abstract of Fraser 2011 (refer to Additional file 2) presents information indicating this is a potential included study, but 80% of students screened this study out.

The primary endpoints of our study were the final sensitivity and screening specificity of citation screening. As with other screening tests, screening sensitivity is of greater importance than specificity to ensure relevant studies are not missed. Students randomised to perform citation screening using ReGroup, a web-based systematic review platform, achieved a significantly higher final sensitivity than those randomised to use EndNote, a widely used desktop reference management program. There were no differences in screening performance between the use of EndNote and either Paper or Screen2Go. The reasons for the improved sensitivity of the ReGroup platform are not clear, but may be related to the design of the user interface, which has been developed to improve the efficiency and experience of systematic review workflow, particularly less experienced users. As would be expected, this improved sensitivity was associated with reduced specificity, suggesting this modality may have lowered participants’ threshold for citation inclusion.

The proportion of participants completing the screening task was numerically higher in the ReGroup and Paper groups. This may be related to the ease of initiation of screening. Login details were emailed to participants randomised to ReGroup, who then simply needed to click on a web link and could immediately commence screening. Similarly, participants randomised to use paper could immediately start screening using the mailed print outs and highlighter pens, whereas those randomised to use EndNote or Screen2Go had to load specific software to their computer or iPhone, respectively.

Time taken to screen was measured across the four intervention groups to compare efficiency between the four modalities. This was similar across the four screening groups although analysis of this outcome is limited by differences in measurement technique. The objectively generated Screen2Go timings were numerically lower than the other three modalities. This may have been due to the improved efficiency of using a phone-based application or over-estimation of screening time with self-report.

There are some limitations to this study. The study was designed as a pilot study and the sample size limits the power of the study to detect small, but relevant differences. The selected study was based on a single Cochrane review update and the two independent expert reviewers had been involved in the original systematic review. Additionally, the medical students had received no prior training and were recruited from a single university. These results may therefore not be generalisable to other forms of review activity, such as full-text review or data extraction or to other groups of potential screeners, such as affected individuals and families. Intention to treat analysis is considered the gold standard in randomised controlled trials to overcome non-compliance and missed outcomes [15]. In our study, primary outcomes are presented as a complete case analysis as this study was a pilot study assessing the feasibility of engaging medical students in citation screening and this approach to analysis is commonly employed in studies of this type.

Future work in this area may assist in investigating ways to optimise the performance of medical students, such as engaging and scalable training, incentives for quality and analytical approaches to deriving the most value from participants’ screening decisions. ‘Crowdsourcing’ citation screening from the general population is another approach to broader participation in systematic review and is an important area for further investigation.

## Conclusions

In summary, the TASER study demonstrated the feasibility of engaging medical students in the screening of citations for systematic reviews. Sensitivity of screening decisions was improved with the use of ReGroup, a web-based systematic review platform, but were otherwise similar across the four modalities studied. Students’ screening performance was modest and highly variable and opportunities exist for improvement with different incentive structures, training and support and alternative analytical approaches. In order to maximise the efficiency of systematic review production, we recommend further investigation into the use of non-expert groups and new technologies for citation screening.

## Competing interests

We declare the following interests: KH is the creator of the mobile screening application, Screen2Go. OC, TT, RG and JHE are the creators of the web-based systematic review program, ReGroup.

## Authors’ contributions

LN and VP participated in the study design, recruitment of study participants and data collection and assisted in the drafting of the manuscript. KH designed the mobile screening application, Screen2Go, and participated in the study’s statistical analysis and drafting of the manuscript. OC and TT designed the web-based systematic review program, ReGroup, and assisted in drafting the manuscript. RG participated in the design of the study and drafting the manuscript. JE conceived the study, participated in its coordination and design and assisted in drafting the manuscript. All authors read and approved the final manuscript.

## Supplementary Material

Additional file 1 **Therapeutic hypothermia in head injury protocol.** All study participants received this file at the commencement of the study to provide background on the systematic review and inclusion criteria for relevant citations. Click here for file

Additional file 2 **Title and abstract of Fraser 2011 and Harris 2009.** The file illustrates the variable amount and clarity of information between citations. Click here for file

## References

[B1] Bastian H Glasziou P Chalmers I Seventy-five trials and eleven systematic reviews a day: how will we ever keep up? PLoS Med 2010 7 9 e1000326 doi:10.1371/journal.pmed.1000326 10.1371/journal.pmed.1000326 20877712 PMC2943439

[B2] Institute of Medicine Finding What Works in Health Care: Standards for Systematic Reviews 2012 Washington: National Academies Press, 2011 Available from: http://www.iom.edu/Reports/2011/Finding-What-Works-in-Health-Care-Standards-for-Systematic-Reviews/Standards.aspx 24983062

[B3] Higgins JPT Green S Cochrane Handbook for Systematic Reviews of Intereventions, Version 5.1.0 [Updated March 2011] 2011 The Cochrane Collaboration Available from: http://www.cochrane-handbook.org

[B4] Edwards P Clarke M DiGuiseppi C Pratap S Roberts I Wentz R Identification of randomized controlled trials in systematic reviews: accuracy and reliability of screening records Stat Med 2002 21 11 1635 1640 10.1002/sim.1190 12111924

[B5] Horton J Vandermeer B Hartling L Tjosvold L Klassen TP Buscemi N Systematic review data extraction: cross-sectional study showed that experience did not increase accuracy J Clin Epidemiol 2010 63 3 289 298 10.1016/j.jclinepi.2009.04.007 19683413

[B6] Reuters T EndNote (software). Version 5 2011

[B7] Clavisi O Turner T Thomas J Cavedon L Development of a Web Based Software Tool to Improve Efficiency and User Experience of Systematic Reviews 2012 Auckland, New Zealand: Proceedings of the 20th Cochrane Colloquium

[B8] Huckvale K van Valthoven M Car J Screen2Go: A Pilot Smartphone App for Citation Screening 2011 Madrid, Spain: Proceedings of the 19th Cochrane Colloquium

[B9] Sydenham E Robertson I Alderson P Hypothermia for traumatic head injury (review) The Cochrane Library 2009 4 51

[B10] Chernoff H Lehmann EL The use of maximum likelihood estimates in χ2 tests for goodness of fit Ann Math Stat 1954 25 3 579 586 10.1214/aoms/1177728726

[B11] Kruskal WH Wallis AW Use of ranks in one-criterion variance analysis J Am Stat Assoc 1952 47 260 583 621 10.1080/01621459.1952.10483441

[B12] Mann HB Whitney DR On a test of whether one of two random variables is stochastically larger than the other Ann Math Stat 1947 18 50 60 10.1214/aoms/1177730491

[B13] Dunn OJ Multiple comparisons among means J Am Stat Assoc 1961 56 293 52 64 10.1080/01621459.1961.10482090

[B14] Spearman C The proof and measurement of association between two rings Amer J Psychol 1904 15 72 101 10.2307/1412159 3322052

[B15] Gupta SK Intention-to-treat concept: a review Perspect Clin Res 2011 2 3 109 112 10.4103/2229-3485.83221 21897887 PMC3159210

