# Supplementary material for: Title and Abstract Screening and Evaluation in Systematic Reviews (TASER): a pilot randomised controlled trial of title and abstract screening by medical students
Source: Syst Rev. 2014 Oct 21;3:121. doi: 10.1186/2046-4053-3-121 (PMC4217707; doi:10.1186/2046-4053-3-121)
Supplement: Additional file 1 — Therapeutic hypothermia in head injury protocol. All study participants received this file at the commencement of the study to provide background on the systematic review and inclusion criteria for relevant citations. [file 2046-4053-3-121-S1.pdf]

THERAPEUTIC HYPOTHERMIA IN HEAD INJURY [PROTOCOL]
(author details have been omitted)

BACKGROUND

Severe traumatic head injury is a major cause of death and disability amongst a predominantly young population, with an estimated 10 million people experiencing such a head injury worldwide every year (Alexander, 1992). There is however, a significant lack of coherent evidence about effective therapies in the acute care of these patients. A recent long-term effort to review the literature and produce management guidelines by the American Association of Neurological Surgeons (Bullock et al, 1996; Kirkpatrick, 1997) could only make four definitive statements about treatment effectiveness which were supported by strong evidence from randomised studies.

Mild to moderate induced hypothermia has been used in the treatment of head injury for over 50 years (Fay, 1945). Although there were several promising experimental studies (Laskowski et al, 1960; Clasen et al, 1968) and case series (Sedzimir, 1959; Shapiro et al, 1974), no controlled clinical studies were performed and the therapy fell from favour. In the last decade, however, several investigators have reported encouraging results of Phase II and III randomised clinical trials (Shiozaki et al, 1993; Clifton et al, 1995; Marion et al, 1997), corroborated by consistent findings of high levels of cerebral protection associated with systemic cooling in well validated laboratory models of global ischemia (Busto et al, 1987). The completed trials are, however, small, single-centre investigations, and do not individually show clear evidence of long-term benefit, except perhaps in subgroups of patients. A larger multi-centre trial is currently underway, but the case for a systematic review of the evidence and a meta-analysis is clear.

Whilst the mechanism of action of such temperature control therapy was originally thought to be primarily a reduction in cerebral metabolic rate (Bering, 1961), there is now evidence that mild hypothermia can also influence the excessive post-traumatic release of excitatory neurotransmitters (Busto et al, 1989), and attenuate the opening of the blood-brain barrier (Smith et al, 1996). The main risks associated with induced systemic hypothermia are an increased risk of sepsis and pneumonia, coagulation abnormalities, and possible myocardial ichaemia and atrial fibrillation (Schubert, 1995).

OBJECTIVES

To determine whether the use of mild therapeutic hypothermia in the treatment of moderate and severe head injury: a) reduces the risk of death (either during the treatment period or at the end of follow up), b) reduces the proportion of patients who at final follow up are either dead or severely disabled, and c) reduces the mean intracranial pressure (ICP) recorded during the treatment period.

CRITERIA FOR CONSIDERING STUDIES FOR THIS REVIEW

Types of studies

All randomised controlled trials of mild hypothermia versus control (open or normothermia) will be included.

Types of participants

Patients with any closed head injury requiring hospitalisation.

Types of interventions

Therapeutic cooling, either locally or systemically, by means of a fluid-filled cooling blanket, a 'bear-hugger' air-cooling device, ice water lavage, any combination of the above, or other methods, to a target temperature of at most 34-35 degrees Celcius for a period of at least 12 hours.
