# Supplementary material for: Title and Abstract Screening and Evaluation in Systematic Reviews (TASER): a pilot randomised controlled trial of title and abstract screening by medical students
Source: Syst Rev. 2014 Oct 21;3:121. doi: 10.1186/2046-4053-3-121 (PMC4217707; doi:10.1186/2046-4053-3-121)
Supplement: Additional file 2 — Title and abstract of Fraser 2011 and Harris 2009. The file illustrates the variable amount and clarity of information between citations. [file 2046-4053-3-121-S2.pdf]

Citation

Author

Fraser, D. D. Close T.E. Rose K.L Ward R. Mehl M. Farrell C. Lacroix J. Creery D. Kesselamn M. Stanimirovic D. Hutchinson J.S. Canadian Critical Care Translational Biology Group

Year

2011

Title

Severe traumatic brain injury in children elevates glial fibrillary acidic protein in cerebrospinal fluid and serum

Abstract

OBJECTIVES: 1) To determine the levels of glial fibrillary acidic protein (GFAP) in both cerebrospinal fluid and serum; 2) to determine whether serum GFAP levels correlate with functional outcome; and 3) to determine whether therapeutic hypothermia as compared with normothermia, alters serum GFAP levels in children with severe traumatic brain injury (TBI)

Citation

Author

Harris O.A, Muh C. R. Surles M. C. Pan Y. Rozycki G. Macleod J. Easley K.

Year

2009

Title

Discrete cerebral hypothermia in the management of traumatic brain injury: A randomized controlled trial - Clinical article

Abstract

Object. Hypothermia has been extensively evaluated in the management of traumatic brain injury (TBI), but no consensus as to its effectiveness has yet been reached. Explanatory hypotheses include a possible confounding effect of the neuroprotective benefits by adverse systemic effects. To minimize the systemic effects, the authors evaluated a selective cerebral cooling system, the CoolSystem Discrete Cerebral Hypothermia System (a "cooling cap"), in the management of TBI. Methods. A prospective randomized controlled clinical trial was conducted at Grady Memorial Hospital, a Level I trauma center. Adults admitted with severe TBI (Glasgow Coma Scale [GCS] score <= 8) were eligible. Patients assigned to the treatment group received the cooling cap, while those in the control group did not. Patients in the treatment group were treated with selective cerebral hypothermia for 24 hours, then rewarmed over 24 hours. Their intracranial and bladder temperatures, cranial-bladder temperature gradient, Glasgow Outcome Scale (GOS) and Functional Independence Measure (FIM) scores, and mortality rates were evaluated. The primary outcome was to establish a cranial-bladder temperature gradient in those patients with the cooling cap. The secondary outcomes were mortality and morbidity per GOS and FIM scores. Results. The cohort comprised 25 patients (12 in the treatment group, 13 controls). There

was no significant intergroup difference in demographic data or median GCS score at enrollment (treatment group 3.0, controls 3.0; p = 0.7). After the third hour of the study, the mean intracranial temperature of the treatment group was significantly lower than that of the controls at all time points except Hours 4 (p = 0.08) and 6 (p = 0.08). However, the target intracranial temperature of 33degree C was achieved in only 2 patients in the treatment group. The mean intracranial-bladder temperature gradient was not significant for the treatment group (p = 0.07) or the controls (p = 0.67). Six (50.0%) of 12 patients in the treatment group and 4 (30.8%) of 13 in the control group died (p = 0.43). The medians of the maximum change in GOS and FIM scores during the study period (28 days) for both groups were 0. There was no significant difference in complications between the groups (p value range 0.20-1.0). Conclusions. The cooling cap was not effective in establishing a statistically significant cranial-bladder temperature gradient or in reaching the target intracranial temperature in the majority of patients. No significant difference was achieved in mortality or morbidity between the 2 groups. As the technology currently stands, the Discrete Cerebral Hypothermia System cooling cap is not beneficial for the management of TBI. Further refinement of the equipment available for the delivery of selective cranial cooling will be needed before any definite conclusions regarding the efficacy of discrete cerebral hypothermia can be reached
